# Epidemiology of Severe Acute Respiratory Illness (SARI) among Adults and Children Aged ≥5 Years in a High HIV-Prevalence Setting, 2009–2012

**DOI:** 10.1371/journal.pone.0117716

**Published:** 2015-02-23

**Authors:** Cheryl Cohen, Sibongile Walaza, Jocelyn Moyes, Michelle Groome, Stefano Tempia, Marthi Pretorius, Orienka Hellferscee, Halima Dawood, Summaya Haffejee, Ebrahim Variava, Kathleen Kahn, Akhona Tshangela, Anne von Gottberg, Nicole Wolter, Adam L. Cohen, Babatyi Kgokong, Marietjie Venter, Shabir A. Madhi

**Affiliations:** 1 Centre for Respiratory Diseases and Meningitis, National Institute for Communicable Diseases of the National Health Laboratory Service, Johannesburg, South Africa; 2 School of Public Health, Faculty of Health Sciences, University of the Witwatersrand, Johannesburg, South Africa; 3 Medical Research Council, Respiratory and Meningeal Pathogens Research Unit, Faculty of Health Sciences, University of the Witwatersrand, Johannesburg, South Africa; 4 Department of Science and Technology/National Research Foundation: Vaccine Preventable Diseases, University of the Witwatersrand, Johannesburg, South Africa; 5 Influenza Division, Centers for Disease Control and Prevention, Atlanta, Georgia, United States of America; 6 Influenza Programme, Centers for Disease Control and Prevention–South Africa, Pretoria, South Africa; 7 Department of Medicine, Pietermaritzburg Metropolitan Hospital and University of KwaZulu Natal, Pietermaritzburg, South Africa; 8 School of Pathology, University of KwaZulu Natal, Pietermaritzburg, South Africa; 9 Department of Medicine, Klerksdorp Tshepong Hospital, South Africa; 10 Department of Medicine, Faculty of Health Sciences, University of the Witwatersrand, Johannesburg, South Africa; 11 MRC/Wits Rural Public Health and Health Transitions Research Unit (Agincourt), School of Public Health, Faculty of Health Sciences, University of the Witwatersrand, Johannesburg, South Africa; 12 Centre for Global Health Research, Umeå University, Umeå, Sweden; 13 INDEPTH Network, Accra, Ghana; 14 Zoonoses Research Unit, Department of Medical Virology, University of Pretoria, Pretoria, South Africa; University of Otago, NEW ZEALAND

## Abstract

**Objective:**

There are few published studies describing severe acute respiratory illness (SARI) epidemiology amongst older children and adults from high HIV-prevalence settings. We aimed to describe SARI epidemiology amongst individuals aged ≥5 years in South Africa.

**Methods:**

We conducted prospective surveillance for individuals with SARI from 2009–2012. Using polymerase chain reaction, respiratory samples were tested for ten viruses, and blood for pneumococcal DNA. Cumulative annual SARI incidence was estimated at one site with population denominators.

**Findings:**

We enrolled 7193 individuals, 9% (621/7067) tested positive for influenza and 9% (600/6519) for pneumococcus. HIV-prevalence was 74% (4663/6334). Among HIV-infected individuals with available data, 41% of 2629 were receiving antiretroviral therapy (ART). The annual SARI hospitalisation incidence ranged from 325-617/100,000 population. HIV-infected individuals experienced a 13–19 times greater SARI incidence than HIV-uninfected individuals (p<0.001). On multivariable analysis, compared to HIV-uninfected individuals, HIV-infected individuals were more likely to be receiving tuberculosis treatment (odds ratio (OR):1.7; 95%CI:1.1–2.7), have pneumococcal infection (OR 2.4; 95%CI:1.7–3.3) be hospitalised for >7 days rather than <2 days (OR1.7; 95%CI:1.2–2.2) and had a higher case-fatality ratio (8% vs 5%;OR1.7; 95%CI:1.2–2.3), but were less likely to be infected with influenza (OR 0.6; 95%CI:0.5–0.8). On multivariable analysis, independent risk indicators associated with death included HIV infection (OR 1.8;95%CI:1.3–2.4), increasing age-group, receiving mechanical ventilation (OR 6.5; 95%CI:1.3–32.0) and supplemental-oxygen therapy (OR 2.6; 95%CI:2.1–3.2).

**Conclusion:**

The burden of hospitalized SARI amongst individuals aged ≥5 years is high in South Africa. HIV-infected individuals are the most important risk group for SARI hospitalization and mortality in this setting.

## Introduction

Pneumonia was the second leading underlying natural cause of death amongst persons aged ≥15 years in South Africa from 2009–2010 and pneumonia is an important cause of morbidity and mortality in HIV-infected adults.[1, 2] There are few published studies estimating the incidence and viral aetiology of severe acute respiratory illness (SARI) amongst older children and adults from high HIV-prevalence settings in Sub-Saharan Africa.[3]

Data on the burden, severity and aetiology of SARI amongst HIV-infected and -uninfected older children and adults are necessary to guide the relative prioritisation of prevention and control efforts. In South Africa, the HIV prevalence amongst individuals aged 15–49 years, the age group with the highest prevalence of HIV, was estimated to be 17% in 2012.[4] South Africa embarked on a national programme of provision of antiretroviral therapy (ART) in 2004.[5] ART coverage amongst eligible HIV-infected adults (CD4+ T cell count<350/mm^3^) in South Africa was estimated to be 29% in 2009 and 52% in 2011.[6]

We aimed to describe the incidence, viral aetiology and factors associated with death amongst HIV-infected and -uninfected individuals aged ≥5 years hospitalised with SARI in South Africa from 2009 through 2012.

## Methods

### Description of the surveillance programme

From February 2009, active, prospective, hospital-based surveillance (the Severe Acute Respiratory Illness (SARI) programme) was implemented in three of the nine provinces of South Africa (Chris Hani-Baragwanath Academic Hospital (CHBAH) in an urban area of Gauteng Province, Edendale Hospital in a peri-urban area of KwaZulu-Natal Province and Matikwana and Mapulaneng Hospitals in a rural area of Mpumalanga Province). In June 2010, an additional surveillance site was introduced at Klerksdorp and Tshepong Hospitals in a peri-urban area of the Northwest Province.

### Case definition

A case of SARI was defined as a hospitalised individual with symptom onset less than seven days prior to admission meeting an adapted World Health Organisation (WHO) case definition for SARI: (1) sudden onset of fever (>38°C) or reported fever, (2) cough or sore throat, and (3) shortness of breath, or difficulty breathing.[7]

### Study procedures

All patients admitted during Monday through Friday were eligible, except for adult patients at CHBAH where enrolment occurred for two of every five working days (enrolment days varied systematically according to the intake days of the two participating wards) per week due to large patient numbers and limited resources. Daily numbers of patients admitted, numbers screened, numbers meeting study case definitions and numbers enrolled were collected in study logs. Study staff completed case report forms until discharge and collected nasopharyngeal (NP) and throat swabs as well as blood specimens for pneumococcal testing from consenting patients. Hospital and ICU admission and collection of specimens for CD4+ T-cell counts was performed at the discretion of the attending-physician. Underlying medical conditions were defined as documented presence of asthma, other chronic lung disease, chronic heart disease, liver disease, renal disease, diabetes mellitus, immunocompromising conditions (excluding HIV infection) or neurological disease.

### Laboratory methods

NP and throat swabs were transported in a single viral transport medium tube at 4–8°C to the National Institute for Communicable Diseases (NICD) within 72 hours of collection. Respiratory specimens were tested by a multiplex real-time reverse-transcription polymerase chain reaction (PCR) assay for influenza A and B viruses, parainfluenza virus 1–3, respiratory syncytial virus (RSV), enterovirus, human metapneumovirus (hMPV), adenovirus and human rhinovirus.[8] Influenza positive specimens were subtyped using the US Centers for Disease Control and Prevention (CDC) real-time reverse-transcription PCR protocol for characterisation of influenza virus. *Streptococcus pneumoniae* was identified by quantitative real-time PCR detecting the *lytA* gene from whole blood specimens.[9] The focus of the surveillance programme was viral pathogens and pneumococcus, therefore patients were not systematically tested for tuberculosis or other respiratory pathogens.

### Evaluation of HIV sero-status

HIV-infection status data was obtained based on testing undertaken as part of standard-of-care,[10] or through anonymised linked dried blood spot specimen testing by enzyme-linked immunosorbent assay (ELISA) in patients providing written informed consent. Results from anonymised testing were used preferentially if both standard-of-care and anonymised results were available. CD4+ T-cell counts were determined by flow cytometry.[11] Patients were categorised into two immunosuppression categories: (1) moderate immunosupression (CD4+ T-lymphocytes ≥200/mm^3^), or (2) severe immunosuppression (CD4+ T-lymphocytes <200/mm^3^).[12] Patients diagnosed by clinicians as HIV-infected on the current admission were referred for HIV management as part of routine care.

### Calculation of incidence

Calculation of incidence was conducted at one surveillance site (CHBAH) where population denominator data were available. This hospital is the only public hospital serving a community of about 1.8 million persons aged ≥5 years in 2012 amongst whom ~10% have private medical insurance.[13] The vast majority (>80%) of uninsured individuals and approximately 10% of medically-insured individuals seek care at public hospitals, consequently the majority of individuals requiring hospitalisation from this community are admitted to CHBAH. We estimated the total number of SARI hospitalisations from the number of enrolled individuals adjusting for non-enrollment in three of five adult wards and during weekends and refusal to participate using information from study logs. The total number of SARI hospitalizations at CHBAH was obtained using the following formula:

$$SARI_{Total}{}_{ij}=SARI_{Enrolled}{}_{ij}*\left(5/2\right)*(7/5)*\left(1/X_{ij}\right)$$ 1

Where *SARI*_*Total*_*ij*__ is the estimated total number of SARI hospitalization in year *i* (2009–2012) and age group *j* (5–14, 15–24, 25–44, 45–64 and ≥65 years of age); *SARI*_*Enrolled*_*ij*__ is the number of SARI cases enrolled in year *i* and age group *j*; 5/2 is the coefficient used to adjust for enrolment of patients in 2/5 adult wards; 7/5 is the coefficient used to adjust for non-enrolment over weekends; and *X*_*ij*_ is the proportion of all eligible cases that were enrolled in year *i* and age group *j*. The adjustment factor varied from 2.2 to 7.9 depending on the age-group and year of enrolment. We estimated incidence of SARI hospitalisations per 100,000 individuals by age groups and HIV status using the adjusted number of SARI hospitalisations divided by the mid-year total population estimates for each year, multiplied by 100,000.[14] HIV prevalence in the study population was estimated from the projections of the Actuarial Society of South Africa AIDS and Demographic model.[4] For estimation of incidence, we assumed that the HIV prevalence by age group amongst patients not tested for HIV was the same as that amongst those tested.

Confidence intervals for incidence estimates were calculated using the Poisson distribution. Age-specific and overall age-adjusted relative risk of SARI hospitalisation in HIV-infected compared to -uninfected persons was determined using log-binomial regression. To explore the possible effect of missing data on estimates of hospitalisation incidence by HIV status, we conducted a sensitivity analysis in which all cases not tested for HIV were assumed to be HIV uninfected.

### Analysis of factors associated with HIV sero-status and death

Univariable and multivariable analyses were performed with Stata version 12 (StataCorp Limited, College Station, United States). To identify factors associated with HIV-infection status and death among SARI patients we implemented multivariable logistic regression models, starting with all variables that were significant at p<0.1 on univariable analysis and dropping non-significant factors with stepwise backward selection. All pairwise interactions of factors significant at the final multivariable additive model were evaluated. Two-sided p-values <0.05 were considered significant. For each univariable analysis, we used all available case information. In the multivariable model, patients with missing data for included variables were dropped from the model. Age group, duration of hospitalisation and year of admission were defined as categorical variables in multiple levels. All other variables were defined as the presence or absence of the attribute excluding missing data. To explore possible bias, individuals tested for HIV were compared to those not tested.

### Ethical considerations

The protocol was approved by the Research Ethics Committees of the Universities of the Witwatersrand and KwaZulu-Natal. This surveillance was deemed non-research by the U.S. CDC and did not need human subjects review by that institution. Written informed consent was obtained from all participants.

## Results

### Demographic, clinical characteristics and aetiology

**Fig 1:**
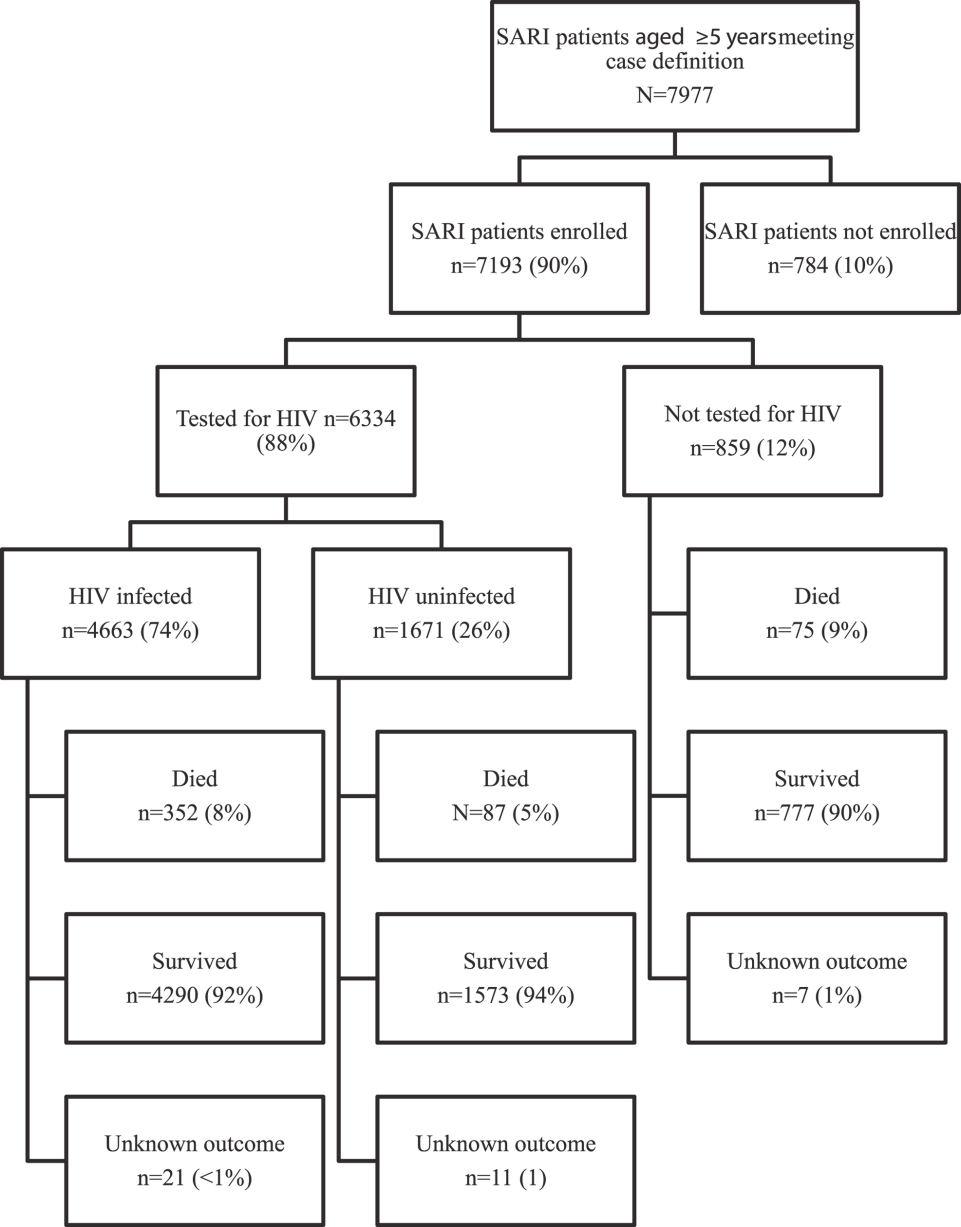
Flow chart of patients aged ≥5 years included in the study. SARI—severe acute respiratory illness, HIV—human immunodeficiency virus.

From February 2009 through December 2012, 7977 individuals ≥5 years of age who fulfilled the SARI case definition were screened for study enrolment, of whom 7193 (90%) were enrolled (Fig. 1). The most common reasons for non-enrolment were being confused or too ill to consent (55%) and study refusal (11%). Of the 7193 enrollees, 8% were 5–14 years of age, 8% 15–24 years, 53% 25–44 years, 25% 45–64 years and 6% ≥65 years (Table 1). The majority of subjects were enrolled at CHBAH (76%), and 61% were female. Among patients with available information, the overall case-fatality ratio was 7%.

**Table 1:** Comparison of the clinical and epidemiologic characteristics of HIV-infected and -uninfected individuals aged ≥5 years hospitalised with severe acute respiratory illness (SARI) at four sentinel surveillance sites, South Africa, 2009–2012.

Characteristics		All patients n/N (%)	HIV-infected n/N (%)	HIV-uninfected n/N (%)	Univariable analysis†		Multivariable analysis††	
					OR(95% CI)	p	OR (95% CI)	p
**Demographic characteristics**								
Age group (years)	5–14	579/7193 (8)	191/4663 (4)	185/1671 (11)	Reference	<0.001	Reference	<0.001
	15–24	599/7193 (8)	336/4663 (7)	192/1671 (11)	1.7 (1.3–2.2)		1.1 (0.8–1.6)	
	25–44	3784/7193 (53)	3016/4663 (65)	405/1671 (24)	7.2 (5.7–9.1)		5.4 (4.1–7.2)	
	45–64	1778/7193 (25)	1047/4663 (22)	564/1671 (34)	1.8 (0.4–2.3)		1.6 (1.2–2.1)	
	≥65	453/7193 (6)	73/4663 (2)	325/1671 (19)	0.2 (0.2–0.3)		0.2 (0.1–0.3)	
Female		4413/7193 (61)	3037/4663 (65)	891/1671 (53)	1.6 (0.5–1.87)	<0.001	1.7 (1.5–2.0)	<0.001
Black African race		6998/7185 (97)	4597/4659 (99)	1573/1670 (94)	4.5 (3.3–6.3)	<0.001	3.8 (2.6–5.6)	<0.001
**Underlying medical conditions**								
Underlying medical condition excluding tuberculosis and HIV*		879/7191 (12)	345/4663 (7)	433/1671 (26)	0.2 (0.2–0.3)	<0.001	0.3 (0.2–0.4)	<0.001
Underlying tuberculosis (receiving tuberculosis treatment on admission)		276/7167 (4)	217/4646 (5)	28/1668 (2)	2.9 (1.9–4.3)	<0.001	2.1 (1.3–3.2)	0.002
Alcohol use		1175/7174 (16)	729/4650 (16)	344/1667 (21)	0.7 (0.6–0.8)	<0.001	0.6 (0.5–0.7)	<0.001
Smoking		1029/7175 (14)	625/4651 (13)	310/1667 (19)	0.7 (0.6–0.8)	<0.001		
**Infectious agents identified**								
Pneumococcus**		600/6519 (9)	499/4506 (11)	70/1601(5)	2.7 (2.1–3.5)	<0.001	2.2 (1.6–2.9)	<0.001
Influenza (any type)		621/7067 (9)	350/4609 (8)	185/1650 (11)	0.7 (0.5–0.8)	<0.001	0.6 (0.5–0.8)	<0.001
Influenza A		366/7067 (5)	190/4609 (4)	113/1650 (7)	0.6 (0.5–0.7)	<0.001		
Influenza B		246/7067 (3)	153/4609 (3)	70/1650 (4)	0.8 (0.6–1.0)	0.083		
Parainfluenzavirus 2		43/7052 (1)	31/4610 (1)	3/1636 (<1)	3.7 (1.1–12.1)	0.031		
Any virus identified***		2279/7056 (32)	1507/4608 (33)	473/1640 (29)	1.2 (1.1–1.4)	0.004		
**Clinical presentation and course**								
Symptoms ≥2 days prior to admission		5934/7059 (84)	3998/4576 (87)	1296/1636 (79)	1.8 (1.6–2.1)	<0.001	1.6 (1.3–1.9)	<0.001
Admission to intensive care		11/7165 (<1)	7/4650 (<1)	2/1665 (<1)	1.3 (0.3–6.0)	0.778		
Mechanical ventilation		11/7167 (<1)	5/4651 (<1)	3/1666 (<1)	0.6 (0.1–2.5)	0.480		
Oxygen required		2682/7164 (37)	1788/4649 (38)	641/1666 (38)	1.0 (0.9–1.1)	0.991		
Antibiotics prescribed on admission		6787/7002 (97)	4468/4569 (98)	1549/1630 (95)	2.3 (1.7–3.1)	<0.001	2.5 (1.7–3.6)	<0.001
Duration of hospitalisation (days)	<2	525/7092 (7)	208/4605 (5)	158/1647 (10)	Reference	<0.001	Reference	
	2–7	4014/7092 (57)	2580/4605 (56)	1016/1647 (62)	1.9 (1.5–2.4)		1.6 (1.2–2.1)	
	>7	2553/7092 (36)	1817/4605 (39)	473/1647 (29)	2.9 (2.3–3.7)		2.4 (1.8–3.2)	
Case-fatality ratio		514/7154 (7)	352/4642 (8)	87/1660 (5)	1.5 (1.2–1.9)	0.001	1.6 (1.2–2.2)	0.002

OR—Odds ratio, CI—confidence interval, HIV—human immunodeficiency virus, CHBAH—Chris Hani Baragwanath Academic Hospital

† HIV-infected vs uninfected

†† HIV-infected vs uninfected. Odds ratios and p values shown for all variables included in the multivariable model

* Asthma, other chronic lung disease, chronic heart disease (valvular heart disease, coronary artery disease, or heart failure excluding hypertension), liver disease (cirrhosis or liver failure), renal disease (nephrotic syndrome, chronic renal failure), diabetes mellitis, immunocompromising conditions excluding HIV infection (organ transplant, immunosuppressive therapy, immunoglobulin deficiency, malignancy), neurological disease (cerebrovascular accident, spinal cord injury, seizures, neuromuscular conditions) or pregnancy. Comorbidities were considered absent in cases for which the medical records stated that the patient had no underlying medical condition or when there was no direct reference to that condition.

**Positive on *lytA* PCR

***Infection with at least one of influenza, parainfluenza virus 1, 2 and 3; respiratory syncytial virus; enterovirus; human metapneumovirus; adenovirus; rhinovirus in addition to influenza

HIV-infection status was available for 6334 (88%) of enrolled individuals. Age-specific HIV prevalence findings were not significantly different when only patients tested through anonymised linked testing were included (data not shown). When comparing patients tested for HIV to those not tested for HIV, controlling for year of test, surveillance site and age group there were no differences in patient epidemiologic characteristics or case-fatality ratios (data not shown). The overall HIV prevalence among persons ≥5 years with available data was 74% (4663/6334) and was highest in the 25–44 year age group (88%, 3016/3421) (Table 1). Twelve percent of individuals had an underlying medical condition, excluding HIV. 53 women were pregnant. Only 14 individuals reported having been vaccinated against influenza in the current year and no subject had received pneumococcal vaccines.

**Fig 2:**
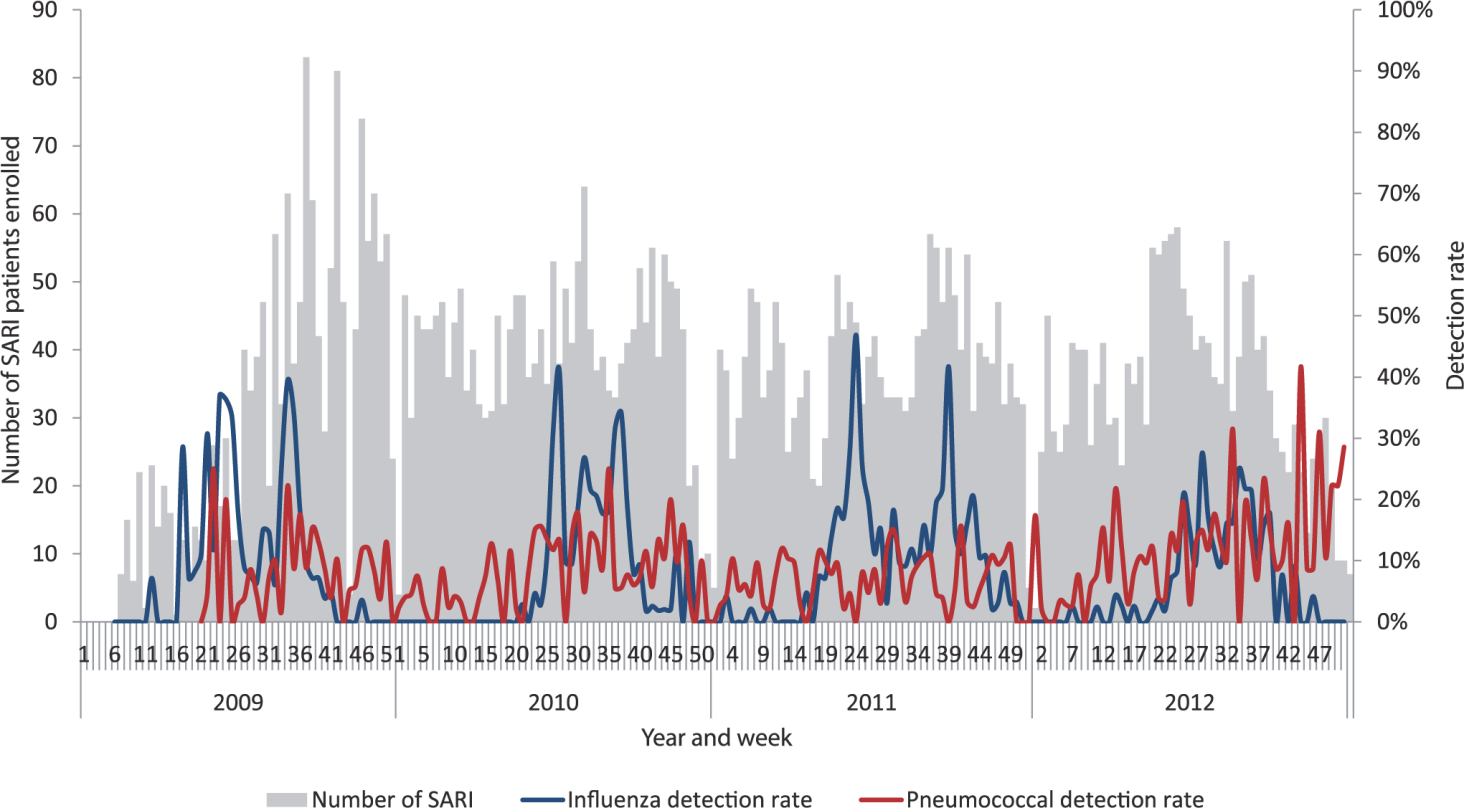
Number of patients enrolled with SARI and influenza, pneumococcal and respiratory syncytial virus (RSV) detection rates by epidemiologic week and year at four sentinel surveillance sites, South Africa, 2009–2011.

Enrolment occurred throughout the year and peaked in the winter months (May-August) (Fig. 2). Overall, among those tested for respiratory viruses, 18% were positive for rhinovirus, 10% for adenovirus and 9% for influenza (Table 2). Other respiratory viruses tested positive in less than 5% of individuals. Adenovirus, rhinovirus and enterovirus were more commonly identified in individuals 5–14 years old than other age groups. Also, 9% of subjects tested positive for pneumococcus on PCR of whole blood specimens. The detection of influenza virus-associated SARI peaked during the winter months (Fig. 2). Although pneumococcus (on *lytA* PCR or culture) was detected perennially, detection increased during winter-months of at least two years (2009 and 2010).

**Table 2:** Percentage of patients testing positive for viral and bacterial pathogens by age group amongst individuals aged ≥5 years hospitalised with severe acute respiratory illness (SARI) at four sentinel surveillance sites, South Africa, 2009–2012.

Age group (years)	5–14 n/N (%)	15–24 n/N (%)	25–44 n/N (%)	45–64 n/N (%)	≥65 n/N (%)	All ages	p*
Influenza	64/560 (11)	64/590 (11)	306/3715 (8)	139/1756 (8)	48/446 (11)	621/7067 (9)	0.010
Adenovirus	115/489 (24)	49/520 (9)	286/3403 (8)	139/1628 (8)	25/413 (6)	613/6453 (10)	<0.001
Enterovirus	43/550 (8)	12/585 (2)	40/3715 (1)	17/1756 (1)	7/446 (2)	119/6933 (2)	<0.001
Rhinovirus	189/550 (34)	128/585 (22)	652/3715 (18)	249/1756 (14)	49/446 (11)	1267/7049 (18)	<0.001
Human metapneumovirus	13/550 (2)	9/585 (2)	68/3715 (2)	26/1756 (1)	8/446 (2)	124/7052 (2)	0.694
Parainfluenzavirus 1	3/550 (1)	2/585 (<1)	11/3715 (<1)	9/1756 (1)	3/446 (1)	28/7052 (<1)	0.599
Parainfluenzavirus 2	8/550 (1)	4/585 (1)	25/3715 (1)	6/1756 (<1)	0/446 (0)	43/7052 (1)	0.021
Parainfluenzavirus 3	7/550 (1)	17/585 (3)	67/3715 (2)	27/1756 (2)	9/446 (2)	127/7050 (2)	0.220
Respiratory syncytial virus	36/550 (7)	21/585 (4)	171/3715 (5)	77/1756 (4)	16/446 (4)	321/7052 (5)	0.118
Any respiratory viral infection	315/550 (57)	209/585 (36)	1159/3715 (31)	491/1756 (28)	105/446 (24)	2279/7056 (32)	<0.001
Infection with >1 respiratory virus	108/550 (19)	47/585 (8)	211/3715 (6)	85/1756 (5)	19/446 (4)	470/7056 (7)	<0.001
Pneumococcus**	24/381 (6)	48/553 (9)	348/3507 (10)	166/1655 (10)	14/423 (3)	600/6519 (9)	<0.001

*chi squared test

**On *lyt*A PCR

### Incidence of hospitalisation in HIV-infected and -uninfected patients

The annual incidence of hospitalisation (per 100,000) for SARI at CHBAH ranged between 325 (95% CI 315–335) in 2012 and 617 (95% CI 603–632) in 2010 and was highest in the 45–64 year age-group; annual range 501 to 1284 (Table 3). HIV-infected individuals experienced an age-adjusted increased relative risk of 13 to 19 times for SARI hospitalisation compared to HIV-uninfected individuals. On sensitivity analysis, assuming that all patients not tested for HIV were HIV-uninfected, the trend towards a higher incidence of SARI hospitalisations in HIV-infected individuals remained in all age groups and years.

**Table 3:** Incidence of severe acute respiratory illness (SARI) hospitalisations per 100,000 population by year and HIV status at Chris Hani-Baragwanath Hospital, South Africa.

Year	Age group (years)	IR (95% CI) All patients	IR (95% CI) HIV infected	IR (95% CI) HIV uninfected	RR (95% CI) HIV infected vs HIV uninfected	RR (95% CI) HIV infected vs HIV uninfected sensitivity analysis∫
2009	5–14	126 (112–141)	1833 (1496–2227)	82 (71–96)	22.1 (17.2–28.4)	6.9 (4.8–9.6)
	15–24	283 (261–308)	2005 (1806–2223)	110 (95–126)	18.2 (15.3–21.8)	12.3 (10.4–14.5)
	25–44	846 (818–875)	2947 (2845–3053)	101 (90–114)	29.0 (25.8–32.8)	11.8 (10.8–12.8)
	45–64	925 (882–970)	4682 (4403–4973)	400 (370–432)	11.7 (10.6–12.9)	8.7 (7.9–9.5)
	≥65	624 (562–690)	8777 (6488–1152)	544 (487–608)	16.1 (11.7–21.7)	12.1 (8.5–16.9)
	All (≥5 years)	591 (577–606)	3072 (2985–3162)	179 (171–188)	18.1 (17.0–19.3)*	10 (9.8–11.0)*
2010	5–14	65 (56–76)	876 (670–1126)	42 (35–52)	20.5 (14.7–28.4)	8.4 (5.5–12.5)
	15–24	206 (187–226)	1665 (149–1858)	67 (56–79)	24.9 (20.3–30.6)	17.5 (14.5–21.3)
	25–44	753 (727–779)	2576 (248–267)	105 (95–117)	24.3 (21.8–27.3)	13.5 (12.4–14.8)
	45–64	1284 (1237–1334)	7025 (671–735)	456 (426–488)	15.4 (14.2–16.7)	12.4 (11.4–13.4)
	≥65	1101 (1022–1185)	19793 (16749–23169)	878 (808–954)	22.5 (18.7–26.9)	20.5 (16.9–24.6)
	All (≥5 years)	617 (603–632)	3175 (3091–3262)	194 (186–203)	19.3 (18.2–20.4)*	13.6 (12.9–14.3)*
2011	5–14	36 (29–44)	376 (252–541)	25 (20–33)	14.4 (9.0–22.7)	9.4 (5.5–15.5)
	15–24	150 (134–167)	998 (859–115)	74 (63–87)	13.4 (10.8–16.7)	12.6 (10.1–15.6)
	25–44	588 (566–611)	1914 (1837–1996)	117 (106–130)	16.3 (14.7–18.3)	14.4 (13.0–16.0)
	45–64	641 (608–677)	3056 (2854–3269)	282 (259–308)	10.8 (9.7–12.1)	10 (9.0–11.2)
	≥65	419 (372–470)	2490 (1564–3633)	389 (344–440)	6.3 (3.9–9.5)	6 (3.7–9.2)
	All (≥5 years)	389 (378–401)	1934 (1869–2001)	134 (127–141)	13.1 (12.2–14.0)*	11.9 (11.1–12.7)*
2012	5–14	33 (26–41)	285 (186–431)	25 (19–32)	11.6 (6.9–18.8)	5.9 (3.1–10.6)
	15–24	134 (119–149)	1154 (1002–1323)	48 (40–59)	23.8 (18.7–30.5)	14.6 (11.6–18.4)
	25–44	505 (485–527)	1665 (1592–1741)	94 (84–106)	17.6 (15.6–19.9)	8.9 (8.1–9.8)
	45–64	501 (472–532)	2448 (2271–2635)	203 (184–225)	12 (10.6–13.6)	9 (8.0–10.2)
	≥65	337 (296–381)	5260 (4052–6692)	252 (217–291)	20.8 (15.4–27.8)	16.6 (12.1–22.5)
	All (≥5 years)	325 (315–335)	1703 (1642–1766)	99 (93–105)	15.8 (14.7–17.1)*	9.6 (8.9–10.3)*

IR—incidence rate, RR—relative risk, CI—confidence interval, HIV—human immunodeficiency virus

Significant relative risk value at p<0.05 are in bold ∫Assuming that all patients not tested for HIV are HIV negative

*Age-adjusted

### Characteristics of HIV-infected patients and factors associated with HIV infection

Compared to HIV-uninfected cases, using multivariable analysis, in addition to other factors, HIV-infected subjects were more likely to be receiving tuberculosis treatment at admission (OR 1.7; 95%CI: 1.1–2.7), have pneumococcal infection (OR 2.4; 95%CI: 1.7–3.3), be hospitalised for >7 days (OR 1.7; 95%CI: 1.2–2.3 as compared to <2 days), and had a higher case-fatality ratio (OR1.7; 95%CI: 1.2–2.3; Table 1). In contrast, HIV-infected subjects were less likely to have an underlying medical condition (OR 0.3; 95%CI: 0.2–0.3), or be infected with influenza (OR 0.6; 95%CI: 0.5–0.8).

Only 1455 (31%) of 4663 HIV-infected patients had available CD4+ T cell count data, of whom 68% (987) had CD4+ T-lymphocyte cell counts <200/mm3. The case-fatality ratio was significantly higher in HIV-infected subjects with severe immunosuppression (12%, 117/983) than those with CD4+ T-lymphocyte count of >200/mm3 (5%, 22/462, p<0.001). Of those with available data, 41% (1083/2629) reported currently receiving ART and 34% (1566/4569) reported receiving prophylaxis with trimethoprim-sulfamethoxazole. The case-fatality ratio was similar in individuals receiving (7%, 80/1075) and not receiving ART (vs. 8%, 121/1536, p = 0.681). The proportion of patients with CD4+ T-lymphocyte cell counts <200/mm3 was higher in patients not receiving ART (388/571, 68%) as compared to patients receiving ART (221/392, 56%).

### Factors associated with mortality

The overall case fatality ratio was 7% (514/7154), with a median age of 42 years (interquartile range 23–74) in those who died. The case-fatality ratio was 1.5 times greater amongst HIV-infected (8%) as compared to HIV-uninfected (5%) individuals with SARI (Table 4). On multivariable analysis, independent risk indicators associated with death included increasing age group, HIV infection (OR 1.8 95%CI: 1.3–2.4), receipt of mechanical ventilation (OR 6.5; 95%CI: 1.3–32.0) and receiving supplementary-oxygen therapy (OR 2.6; 95%CI: 2.1–3.2) (Table 4).

**Table 4:** Factors associated with death amongst patients aged ≥5 years hospitalised with severe acute respiratory illness (SARI) at four sentinel surveillance sites, South Africa, 2009–2012†.

Characteristics		Case-fatality ratio (%)	Univariable analysis		Multivariable analysis†	
			OR (95% CI)	p	OR (95% CI)	p
**Demographic characteristics**						
Age group (years)	5–14	12/577 (2)	Reference	<0.001	Reference	<0.001
	15–24	28/594 (5)	2.3 (1.2–4.6)		3.0 (1.2–7.5)	
	25–44	255/3760 (7)	3.4 (1.9–6.2)		3.4 (1.5–7.9)	
	45–64	171/1774 (10)	5.0 (2.8–9.1)		5.9 (2.5–13.7)	
	≥65	48/449 (11)	5.6 (3.0–10.7)		9.1 (3.7–22.2)	
Race	Other race	6/187 (3)	Reference	0.038	Reference	0.033
	Black African	508/6959 (7)	2.4 (1.0–5.4)		3.5 (1.1–11.2)	
Site	CHBAH	371/5424 (7)	Reference	0.016	Reference	0.001
	Matikwana/Mapulaneng	77/948 (8)	1.2 (0.9–1.6)		1.8 (1.3–2.6)	
	Edendale	55/554 (10)	1.5 (1.1–2.0)		1.6 (1.1–2.4)	
	Klerksdorp	11/228 (5)	0.7 (0.4–1.3)		0.9 (0.5–1.8)	
**Underlying medical conditions**						
HIV status	Negative	87/1660 (5)	Reference	0.001	Reference	<0.001
	Positive	352/4642 (8)	1.5 (1.2–1.9)		1.8 (1.3–2.4)	
Underlying medical condition*	No	456/6281 (7)	Reference	0.52		
	Yes	58/871 (6)	0.9 (0.7–1.2)			
Underlying tuberculosis	No	475/6854 (7)	Reference	<0.001	Reference	0.001
	Yes	36/274 (13)	2.0 (1.4–2.9)		2.0 (1.3–3.0)	
**Infectious agents identified**						
Pneumococcus**	No	413/5885(7)	Reference	0.187		
	Yes	50/597 (8)	1.2 (0.9–1.6)			
Influenza	No	473/6413 (7)	Reference	0.014		
	Yes	29/616 (5)	0.6 (0.4–0.9)			
**Clinical presentation and course**						
Duration of symptoms prior to admission	< 2 days	56/1122 (5)	Reference	0.003	Reference	0.039
	≥ 2 days	441/5899 (7)	1.5 (1.2–2.0)		1.4 (1.0–2.0)	
ICU admission	No	511/7133 (7)	Reference	0.01		
	Yes	3/11 (27)	4.9 (1.3–18.4)			
Mechanical ventilation	No	510/7135 (7)	Reference	<0.001	Reference	0.022
	Yes	4/11 (36)	7.4 (2.2–25.4)		6.5 (1.3–32.0)	
Oxygen therapy	No	215/4466 (5)	Reference	<0.001	Reference	<0.001
	Yes	299/2677 (11)	2.5 (2.1–3.0)		2.6 (2.1–3.2)	
Antibiotics prescribed on admission	No	19/215 (9)	Reference	0.349		
	Yes	484/6760 (7)	0.8 (0.5–1.3)			
Duration of hospitalisation (days)	<2	39/523 (7)	Reference	0.002	Reference	<0.001
	2–7	252/4011 (6)	0.8 (0.6–1.2)		0.5 (0.3–0.7)	
	>7	219/2552 (9)	1.2 (0.8–1.7)		0.6 (0.4–1.0)	

OR—Odds ratio, CI—confidence interval, HIV—human immunodeficiency virus, CHBAH—Chris Hani Baragwanath Academic Hospital

†Additional factors evaluated and found to be non-significant on univariable analysis: sex, alcohol, smoking, and infection with adenovirus, enterovirus, rhinovirus, human metapneumovirus, parainfluenza virus 1, 2 and 3 and respiratory syncytial virus

*Asthma, other chronic lung disease, chronic heart disease (valvular heart disease, coronary artery disease, or heart failure excluding hypertension), liver disease (cirrhosis or liver failure), renal disease (nephrotic syndrome, chronic renal failure), diabetes mellitis, immunocompromising conditions excluding HIV infection (organ transplant, immunosuppressive therapy, immunoglobulin deficiency, malignancy), neurological disease (cerebrovascular accident, spinal cord injury, seizures, neuromuscular conditions) or pregnancy. Comorbidities were considered absent in cases for which the medical records stated that the patient had no underlying medical condition or when there was no direct reference to that condition.

**On *lyt*A PCR

## Discussion

More than two thirds (>70%) of individuals aged ≥5 years hospitalised with SARI in South Africa are co-infected with HIV, making this by far the most important underlying risk condition for this syndrome even in the era of widespread availability of ART. HIV-infected individuals had a 13–19 times greater incidence of SARI hospitalisation than HIV-uninfected individuals and also experienced prolonged hospitalisation and increased risk of death. The spectrum of viral infectious agents identified from HIV-infected and -uninfected individuals was generally similar, however HIV-infected individuals were more likely to test positive for pneumococcus.

The overall incidence of SARI hospitalisation ranged from 325–617/100,000, somewhat greater than was described in another high HIV-prevalence setting in Kenya (229/100,000).[15] The incidence of SARI hospitalisation in HIV-uninfected individuals aged ≥5 years ranged from 99–194/100,000 population each year, similar to what has been described from low HIV-prevalence middle income countries such as Bangladesh (110–130/100,000) and Thailand (incidence in all ages 177–580/100,000) and slightly lower than the incidence in US adults (267/100,000).[16–18] Differences in incidence observed in different settings may be related to differences in health-seeking behavior, differing thresholds for hospital admission and case definitions or may reflect real differences. The 13–19 times elevated incidence (1703–3175/100,000) of hospitalised SARI which we observed in HIV-infected individuals was somewhat greater than the 4 times elevated incidence described in HIV-infected adults from Kenya with outpatient and hospitalised ARI.[3] Amongst HIV-infected individuals the peak incidence was in the 25–64 years age group, the age group most affected by HIV. Amongst HIV-uninfected individuals, incidence increased with increasing age, similar to that seen in low HIV-prevalence countries.[18]

We identified at least one respiratory virus in approximately one-third of all patients, similar to other studies from adults.[19, 20] The prevalence of detection of most respiratory viruses was highest in the 5–14 year age-group and decreased with increasing age. Rhinovirus and adenovirus were most commonly detected, followed by influenza. While the detection of influenza virus in persons aged ≥5 years with SARI likely reflects an aetiologic role, the clinical relevance of many of the other respiratory viruses is unclear without a comparison to controls.[3, 19]

Pneumococcus was identified in 9% of individuals overall with the highest detection rate in persons aged 25–64 years, the age group most affected by HIV. While real-time PCR is more sensitive than blood culture for diagnosing pneumococcal SARI, additional cases of pneumococcal co-infection may still have been missed.[21] Healthy adults are rarely colonized with the pneumococcus and previous studies have found the lytA PCR on blood to be negative in healthy children colonized with the pneumococcus [22–24]. For this reason, we feel that detection of this target in the blood of these SARI patients likely serves as a specific marker for pneumococcal disease. Sterile specimen cultures for bacteria were performed uncommonly (<15% of patients) and thus we were not able to compare bacterial culture with PCR results. On multivariable analysis pneumococcus was significantly more likely to be detected in HIV-infected than HIV-uninfected individuals, likely reflecting the very high relative risk of hospitalisation for pneumococcal SARI in HIV-infected adults.[25] Pneumococcal polysaccharide vaccine is used uncommonly in South Africa, but this vaccine is not recommended for HIV-infected adults.[26] Although more recent data suggest that the pneumococcal conjugate vaccine may be effective in HIV-infected adults in Africa, [27] there is no specific recommendation for this vaccine in adults in South Africa. The pneumococcal conjugate vaccine was introduced into the routine childhood immunisation programme in 2009. This may have impacted on the proportion of patients testing positive for pneumococcus over time as a result of indirect protection conferred to unvaccinated adults.[28, 29]

In contrast to pneumococcus, influenza virus was significantly less commonly identified from HIV-infected individuals. We have previously demonstrated, in the same population, that HIV-infected individuals aged 25–44 years have an ~10–20 times increased incidence of hospitalisation for influenza.[30] The relatively lower detection rates in our study likely reflect the fact that HIV-infected individuals have a substantially elevated risk of other important pathogens such as pneumococcus, *Pneumocystis jirovecii* and tuberculosis which contribute to a greater proportion of SARI cases in the HIV-infected, rather than an absolute lower risk in HIV-infected individuals. This has been described for respiratory viral infections in HIV-infected children from South Africa.[31]

The overall case-fatality ratio was 7%, similar to other studies from Africa and the US.[3, 15, 18, 32, 33] Increasing age was a risk factor for death, similar to that observed in developed country settings.[34] However, the median age at death was 42 years (36 years in HIV-infected and 62 years in HIV-uninfected), lower than the median age at death in more developed settings where death is more common in elderly individuals. HIV-infected individuals were 1.5 times more likely to die than HIV-uninfected individuals in contrast to other studies which have found a similar mortality in HIV-infected and -uninfected individuals.[32, 35, 36] Earlier studies included smaller numbers of cases and may have been underpowered to detect the relatively modest increased relative risk of death. In addition, in other studies, HIV-uninfected individuals may have had a higher proportion of elderly or persons with underlying illness than in our study. Receiving tuberculosis treatment on admission was also a risk factor for death. A study in South African gold miners found that underlying lung damage from tuberculosis was a risk factor for SARI mortality.[37] Patients who died had a shorter duration of hospitalisation, suggesting that death occurred early during admission. A longer duration of symptoms prior to hospitalisation was also associated with increased mortality, thus delayed clinical presentation and subsequent delayed treatment initiation may have contributed to mortality in some cases. Mechanical ventilation and supplementary oxygen therapy were independent predicators of mortality. It is likely that these factors are surrogates for disease severity. Unfortunately, data on oxygen saturation were not available.

Approximately 40% of patients with available data reported receiving ART on admission. Suggesting that even in the presence of ART, pneumonia remains a common clinical presentation in HIV-infected individuals. More than two thirds of patients with available data had severe immunosuppression on CD4+ T cell count and a low CD4+ T cell count was associated with increased mortality. Data on receipt of ART and CD4+T cell counts was unfortunately available for less than half of all HIV-infected patients and no data on ART compliance or clinical HIV stage was available potentially biasing results. In addition, data on socioeconomic status of patients were not available.

Additional limitations of our study include that subjects were only tested systematically for ten viruses and pneumococcus. Blood cultures were not performed systematically and we did not test for *P*. *jirovecii* or tuberculosis, important causes of pneumonia in HIV-infected individuals.[36, 38] Our study may have underestimated mortality because severely ill cases may have been less likely to consent to inclusion or may have died before or shortly after hospital admission prior to being consented, as has been previously suggested.[39] Our estimates of incidence were only obtained from one surveillance hospital and assumed that all individuals in the community accessed care at CHBH hospital. In addition, we did not account for individuals who did not seek care at all. Therefore our incidence and mortality estimates likely represent a minimum estimate. Nevertheless, the estimates of relative risk by HIV status should be robust, unless patients had differential access to care by HIV-infection status. Missing information for some of the predictors in our logistic regression model may have resulted in a loss of power that may have potentially hindered our ability to assess significance for some of the predictors assessed in our model and could have potentially introduced bias.

Efforts to promote earlier diagnosis of HIV infection and earlier ART initiation as well as more widespread ART availability may reduce the substantial burden of disease in HIV-infected individuals and improve outcomes in patients with SARI. Pneumococcus and influenza were commonly detected aetiologies. This suggests that more widespread access to vaccination against influenza and pneumococcus as well as indirect protection following the introduction of pneumococcal conjugate vaccine in children in South Africa could also reduce the burden of SARI.
